# Protocol for spatiotemporal analysis of climate-induced negative sentiment in social media

**DOI:** 10.1016/j.xpro.2026.104656

**Published:** 2026-06-23

**Authors:** Tareq Al-Ahdal, Sandra Barman, Barrak Alahmad, Joacim Rocklöv

**Affiliations:** 1 Heidelberg Institute of Global Health, Section for Oral Health, Faculty of Medicine and University Hospital, Heidelberg University, Heidelberg, Germany; 2 Interdisciplinary Center for Scientific Computing (IWR), Heidelberg University, Heidelberg, Germany; 3 Bioeconomy and Health, RISE Research Institutes of Sweden, Gothenburg, Sweden; 4 Department of Environmental Health, Harvard T.H. Chan School of Public Health, Harvard University, Boston, MA, USA; 5 Department of Public Health and Clinical Medicine, Section of Sustainable Health, Umeå University, Umeå, Sweden; 6 Heidelberg Institute of Global Health (HIGH), Heidelberg University Hospital, Heidelberg University, Heidelberg, Germany

**Keywords:** Health Sciences, Computer sciences, Environmental sciences

## Abstract

Climate-induced stressors are associated with population-level mental well-being, yet reproducible workflows linking environmental data to social media sentiment remain scarce. Here, we present a protocol for analyzing spatiotemporal associations between climate-induced stressors and negative sentiment from geotagged social media data. We describe steps for preprocessing large-scale geotagged X data, performing multilingual sentiment analysis with Linguistic Inquiry and Word Count (LIWC-22), integrating climate and health indicators, and fitting a Bayesian spatiotemporal Poisson model. This protocol supports analyses of associations across diverse climate stressors and European regions.

For complete details on the use and execution of this protocol, please refer to Al-Ahdal et al.^1^ and Al-Ahdal et al.^2^

## Highlights

• Protocol for analyzing climate-sentiment associations from geotagged social media
• Multilingual sentiment analysis of large-scale tweets with LIWC-22 across 7 languages
• Bayesian spatiotemporal modeling using INLA with fused-lasso regularization
• Reproducible workflow for integrating climate, health, and social media data

## Abstract (editor highlights)

Publisher’s note: Undertaking any experimental protocol requires adherence to local institutional guidelines for laboratory safety and ethics.

## Graphical abstract

**Figure:**
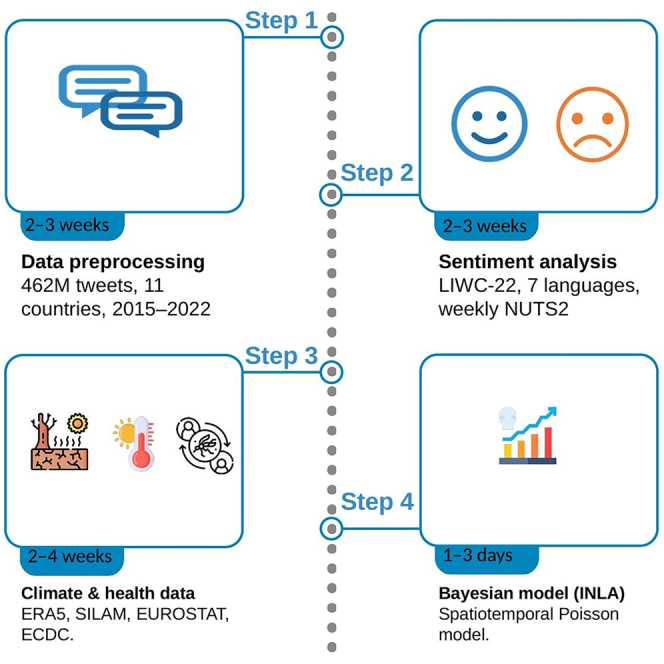

## Teaser

Climate-induced stressors are associated with population-level mental well-being, yet reproducible workflows linking environmental data to social media sentiment remain scarce. Here, we present a protocol for analyzing spatiotemporal associations between climate-induced stressors and negative sentiment from geotagged social media data. We describe steps for preprocessing large-scale geotagged X data, performing multilingual sentiment analysis with LIWC-22, integrating climate and health indicators, and fitting a Bayesian spatiotemporal Poisson model. This protocol supports analyses of associations across diverse climate stressors and European regions.

## Before you begin

This protocol is suitable for researchers investigating climate change impacts on mental wellbeing, public health surveillance using social media, or spatiotemporal epidemiological modelling. Users should have intermediate experience with R and Python, familiarity with geospatial data analysis, and a basic understanding of Bayesian statistical methods. Prior experience with high-performance computing environments is helpful but not required for the modelling steps.

### Institutional permissions

This protocol uses geotagged social media data, which may contain potentially identifiable information even when usernames are removed. Future users should: (a) confirm that the data source permits academic research use; (b) remove user identifiers and metadata before analysis; (c) aggregate results to coarse spatial and temporal units before reporting; and (d) consult their institutional review board.

For the associated research articles, ethical approval was not required as the data was secondary and analyzed only in aggregated form. Local requirements may differ.

### Innovation

This protocol integrates several established techniques into a reproducible workflow for analysing spatiotemporal associations between climate-induced stressors and population-level negative sentiment expressed on social media. Three elements distinguish it from existing approaches. First, it harmonises gridded climate and environmental reanalysis (ERA5, SILAM), public-health indicators (EUROSTAT, ECDC), and large-scale geotagged social-media data on a single NUTS2 weekly grid, an integration not previously documented as a protocol. Second, multilingual dictionary-based sentiment classification (LIWC22) is applied across seven European languages, producing weekly negative-tweet counts directly usable as a Poisson outcome. Third, the modelling step couples indirect standardisation with a Bayesian spatiotemporal Poisson model fitted in R-INLA, combining BYM2 spatial structure, RW1 smoothing, country-level IID effects, and fused-lasso regularisation of temporal change points, with documented adaptations for single-country use.

### Computational environment setup

**Timing: 2 weeks**

1. Install R (version 4.2.2 or higher) and RStudio.
2. Install Python (version 3.11.2 or higher).
3. Install required R packages: readxl, tidyr, dplyr, sf, ggplot2, scales, spdep, terra, exactextractr, dtplyr, lubridate, pals, gridExtra, RColorBrewer, Matrix.
4. Install INLA from the special repository as it is not distributed through the CRAN (https://www.r-inla.org/).Run: install.packages(“INLA”, repos = c(getOption(“repos”), INLA = “https://inla.r-inla-download.org/R/stable”), dep = TRUE). Verify the installation by running library(INLA) followed by inla.version().

***Note:*** INLA installation can fail due to missing system dependencies. On Linux, ensure that the system libraries libgsl, libudunits2, and libgdal are installed before attempting INLA installation. If installation from the stable branch fails, try the testing branch by replacing the URL with https://inla.r-inla-download.org/R/testing. A complete installation script is provided in the GitHub repository (INLAinstall.R).

5. Install required Python libraries:
  a. Data and geographic analysis: pandas, numpy, geopandas, shapely.
  b. Natural language processing: nltk, langdetect, textblob.
  c. Download NLTK resources after install: punkt, stopwords, wordnet, averaged_perceptron_tagger.
6. Obtain a LIWC22 license from https://www.liwc.app/with dictionaries for the languages of interest (this protocol uses English, German, French, Italian, Spanish, Portuguese, and Dutch).

***Note:*** LIWC22 also offers dictionaries for non-European languages. Check the LIWC website for the current list of supported languages.

***Note:*** Complete installation scripts and package lists are provided in the GitHub repository linked in the Resource availability section.

**CRITICAL:** Processing hundreds of millions of tweets requires high-performance computing resources. Recommended specifications: 64+ CPU cores, 400GB+ RAM, and 1TB+ storage. Consult your institutional HPC service.

**CRITICAL:** LIWC22 is a dictionary-based tool and does not strictly require the full natural language processing (NLP) pipeline used by machine-learning sentiment approaches. Light cleaning (removing URLs, @mentions, hashtags, emojis, and non-alphabetic characters) is sufficient. Users who only intend to apply LIWC22 may skip stemming, lemmatization, and stopword removal. The pipeline used in the associated research articles applies the full preprocessing pipeline (Step 1) because alternative machine-learning sentiment models were tested in parallel; users who want to compare LIWC22 results against such models should follow the same approach.

### Data acquisition

**Timing: 1–4 weeks (depending on data access)**

7. Acquire geotagged Twitter/X data from Harvard CGA Geotweet Archive v2.0 (https://gis.harvard.edu/geotweet-archive-v20). The data is available only until July 12, 2023 as the X platform has closed the free Access to API.
8. Download NUTS shapefiles from Eurostat for NUTS2 and NUTS3 regional boundaries. Make sure to use the same NUTS vintage for X data, climatic data and pollens as they might not match in the end.
9. Download climate data as NetCDF files from Copernicus Climate Data Store (ERA5 reanalysis).
10. Download pollen data from SILAM (Finnish Meteorological Institute) for alder, birch, and olive concentrations.
11. Extract climate variables to NUTS3 regions using terra and exactextractr packages in R (mean aggregation). Process to weekly resolution.

***Note:*** Data extraction scripts (europe.R) and processing notebooks (process.ipynb) are available in the GitHub repository.

## Key resources table

**Table:** 

REAGENT or RESOURCE	SOURCE	IDENTIFIER
**Deposited data**		

Geotagged Twitter/X data (Geotweet Archive v2.0)	Harvard Center for Geographic Analysis	https://gis.harvard.edu/geotweet-archive-v20
ERA5 climate reanalysis	Copernicus Climate Data Store	https://cds.climate.copernicus.eu/
SILAM pollen reanalysis (alder, birch, olive)	Finnish Meteorological Institute	https://silam.fmi.fi/
Heat-attributable mortality	EUROSTAT	https://ec.europa.eu/eurostat
West Nile virus incidence	European Centre for Disease Prevention and Control (ECDC)	https://www.ecdc.europa.eu
NUTS shapefiles	Eurostat	NUTS_RG_20 M_2006_4326
Country boundary shapefiles	Natural Earth	ne_110 m_admin_0_countries

**Software and algorithms**		

R Statistical Software (v4.2.2)	R Foundation for Statistical Computing	https://www.r-project.org/
Python (v3.11.2)	Python Software Foundation	https://www.python.org/
R-INLA package (v24.3.9)	Rue et al.^3^	https://www.r-inla.org/
LIWC-22	Boyd et al.^4^	https://www.liwc.app/
terra (R package)	Hijmans^5^	https://CRAN.R-project.org/package=terra
exactextractr (R package)	Daniel Baston	https://CRAN.R-project.org/package=exactextractr
spdep (R package)	Bivand et al.^6^	https://CRAN.R-project.org/package=spdep
genlasso (v1.6.1)	Arnold and Tibshirani^7^	https://CRAN.R-project.org/package=genlasso
Analysis code (this protocol)	Al-Ahdal et al.	https://github.com/alahdalta1/Germany_Climate_Sentiment_Paper, https://doi.org/10.5281/zenodo.19843730

**Other**		

High-performance computing cluster (Linux, 64+ CPU cores, 400 GB+ RAM, 1 TB+ storage)	Institution-specific	N/A

## Materials and equipment

### Hardware requirements

• High-performance computing cluster with 64+ CPU cores and 400GB+ RAM for tweet processing
• Minimum 1TB storage for raw data, climate NetCDF files, and intermediate outputs
• Standard workstation (16GB+ RAM) sufficient for INLA modelling and visualization steps

### Software configuration

• Increase the shell stack-size limit before launching R for large INLA models (Linux: ulimit -s unlimited). This is a shell setting, not an INLA parameter.
• LIWC22 CLI version required for batch processing
• Custom R library path recommended for HPC environments

## Step-by-step method details

### Data preprocessing and text cleaning

**Timing: 2–3 weeks for ∼400 million tweets on our HPC, but this depends on how many cores you can request and how the tweets split by language**

This step prepares raw geotagged tweets for sentiment analysis using Python 3.11.2. It removes noise from the tweet text, detects each tweet’s language, and assigns geographic and temporal indices for downstream aggregation.

***Note:*** Refer to get_NLP15_7.py in the repository for the complete implementation.

1. Perform text preprocessing
  a. Strip URLs.
  b. Strip @mentions.
  c. Strip punctuation and digits.
  d. Lowercase the text.
  e. Drop stopwords with NLTK.
  f. Optionally, apply stem tokens Stem with SnowballStemmer and lemmatize with WordNetLemmatizer, only do this if you also plan to feed the same tweets into an ML sentiment model. For LIWC22 alone, light cleaning is enough (see the CRITICAL note in Before you begin).

**CRITICAL:** LIWC22 looks up exact word forms , if half your tweets are stemmed and half are not, the sentiment scores will not be comparable. Light cleaning of the text is recommended

2. Perform language detection:
  a. Use the platform-supplied tweet_lang field where it is present; fall back to langdetect when it is not.
  b. Keep only the seven languages LIWC22 supports here: English, German, Spanish, Portuguese, Dutch, French, Italian.
3. Perform geographic assignment: Spatial-join each tweet’s coordinates against the Natural Earth country boundaries shapefile using geopandas to get a country code per tweet.
4. Perform temporal indexing: With lubridate, pull year, month, and ISO week number from each tweet’s timestamp, then compute weeks elapsed since your study start date (e.g., 2015-01-01). This gives you a single integer week index that the model in Step 5 will use.

***Note:*** Pick a file-naming scheme on day one and keep to it. Something like tweets_DE_2019_cleaned_v2.csv — country, year, processing stage, version. We learned this the hard way: with hundreds of intermediate files across countries, languages, and re-runs, untraceable filenames are by far the most common cause of bugs.

### Sentiment analysis using LIWC-22

**Timing: 2–3 weeks for 400 million tweets**

This step assigns each tweet a sentiment label and aggregates the results to weekly counts per NUTS2 region, using the LIWC22 command-line interface driven by a Python batch script.

***Note:*** Refer to the_code_for_LIWC_CLI.py and testRbase.R in the repository for the complete implementation.

5. Process tweets through LIWC22 command-line interface for each language, extracting posemo (positive emotion) and negemo (negative emotion) scores.
6. Classify each tweet by sentiment:
  a. Classify each tweet using the LIWC22 posemo (positive emotion) and negemo (negative emotion) scores:
    i. posemo > negemo: label tweet as positive.
    ii. negemo > posemo: label tweet as negative.
    iii. posemo = negemo = 0: label tweet as neutral.
  b. For detailed analysis, additionally track the categories only_positive, only_negative, and both_positive_and_negative.

***Note:*** The classification of tweets into positive, negative, and neutral categories is intentional and methodologically motivated. The downstream analysis aggregates to weekly counts of negative tweets per NUTS2 region (item 7), which serves as the outcome in a Poisson regression model (Step 5). We needed counts, so each tweet had to be placed in one category so if a tweet’s negemo score was higher than its posemo score we labelled it negative this gives a clear decision for every tweet and kept the classification easy to interpret.

7. Aggregate classified tweets to weekly counts: Once tweets have been classified, group them by NUTS region and ISO week then calculate NTw (negative tweet count) and TTw (total tweet count) for each region-week combination.

**Pause point:** This is a good place to stop and save your output after weekly aggregation, the resulting region-week sentiment count file (NTw and TTw per NUTS2 region per week) can be saved.Subsequent steps (Climate and health data integration; Bayesian modelling) read this file as input and do not require Steps 1–2 to be re-run.

### Climate and health data integration

**Timing: 2–4 weeks for climate pollen data processing**

This step builds the exposure dataset by extracting gridded climate, pollen, and health indicators and aligning them to the NUTS2 weekly grid, using R 4.2.2 (terra, exactextractr) and Python 3.11.2 (process.ipynb).

***Note:*** Refer to europe.R and process.ipynb in the repository for complete implementation.

8. Extract climate variables from ERA5 reanalysis:
  a. Read the NetCDF files with the terra package.
  b. Using extactextracr, aggregate raster values to NUTS3 regions taking mean aggregation across the cells.
  c. Apply the process to each of the variables: temperature (mean, max, min), precipitation, wind speed, wind direction, solar radiation, cloud fraction, and relative humidity.
  d. Resample the resulting time series to weekly resolution.

**CRITICAL:** Use the same NUTS vintage (e.g., 2006, 2010, 2013, 2016, or 2021) across Twitter, climate, pollen, and health datasets. Region codes and boundaries change between vintages, and mismatched vintages produce silent join failures, missing regions, and incorrect spatial aggregations. Verify all region codes against a single reference shapefile before proceeding.

9. Integrate additional climate and health exposure variable: add the remaining exposure variables to the dataset, Integrate SPI (Standardized Precipitation Index) for drought, pollen concentrations (alder, birch, olive), heat-attributable mortality (estimates modelled from EUROSTAT 2015–2019 mortality data, extended to 2015–2022), and West Nile virus incidence (ECDC). The key resources table lists each source.

***Note:*** This protocol is not restricted to these particular variables, it can be used with other climate or environmental stressors beyond those listed above. Any variable available as gridded or regional time-series data can be processed using the same extraction and aggregation steps.

***Note:*** The Standardized Precipitation Index (SPI) was selected over alternatives such as SPEI (Standardized Precipitation Evapotranspiration Index) or the Palmer Drought Severity Index because it requires only precipitation as input, making it directly computable from ERA5 reanalysis data with consistent coverage across all study regions.

***Note:*** The choice of alder, birch, and olive pollens were selected based on the SILAM reanalysis dataset, which provides consistent gridded weekly concentrations for these three taxa across Europe from 2015 onwards (Sofiev et al.^8^). These taxa also rank among the most clinically relevant aeroallergens in Europe and span complementary flowering windows: alder and birch in late winter and spring, olive in early summer. Grass and ragweed pollens were not included because gridded reanalysis coverage was less complete during the study period; users with access to other pollen reanalysis can integrate additional taxa using the same workflow.

10. Bin continuous climate variables for non-linear modelling with RW1 random effects:
  a. Compute bin thresholds from the pooled distribution of climate values across all study regions.
  b. Convert each continuous climate variable into categorical bins using the derived thresholds.
  c. Verify that bin categories are consistent across all NUTS2 regions before modelling.

**CRITICAL:** Bin thresholds for continuous climate variables are study-specific. Thresholds reported in the associated research articles were derived from the European NUTS2 weekly distribution and should not be applied to data from different geographies or time periods without re-derivation. Using inappropriate thresholds yields misleading exposure–response patterns.

***Note:*** The implication that, for example, 30 °C is “extreme” everywhere is mitigated by two design features of the protocol: (a) region-specific spatial random effects and country-level IID effects (Step 14) absorb baseline differences in regional sentiment that would otherwise confound exposure effects; and (b) RW1 random effects smooth abrupt jumps between adjacent bins. A sensitivity analysis comparing four-bin and three-bin categorisations was reported in Al-Ahdal et al.^2^ (Supplementary Tables S3–S5) and showed that exposure–response patterns remained consistent in both direction and magnitude. Users for whom region-specific exposure thresholds are essential (e.g., physiological adaptation studies) may implement region-specific binning by computing quantile thresholds within each NUTS2 region rather than across the pooled dataset, and assigning each tweet to its region-specific bin before modelling. However, this approach should be applied cautiously: when the number of observations within a region is small, region-specific quantile thresholds may be unstable and sensitive to outliers, potentially producing bin boundaries that do not generalize well.

11. Apply fused lasso regularisation to identify temporal change points:
  a. Run fused lasso regularisation on each country’s time series using the genlasso package.
  b. Generate country-specific β coefficients (β_1_ through β_K) representing K piecewise-constant temporal intervals.

***Note:*** When the protocol is applied to a single-country dataset, the country index reduces to a constant (c(i) = 1 for all i) and the country-specific β coefficients become a single global piecewise-constant temporal effect. In that case, an RW1 effect on the week index (Step 14) can serve as a simpler alternative, bearing in mind that RW1 produces a smoothly varying temporal effect rather than the exact change points and piecewise-constant segments enforced by the fused lasso's sparsity penalty, so the two are not strictly equivalent. The appropriate choice depends on whether discrete regime shifts or smooth variation is expected.

### Bayesian spatiotemporal modeling with INLA

**Timing: Approximately 10 min to fit a single model on the cluster and 1–3 days to fit the full set of models on the European NUTS2 weekly dataset, depending on hardware. Model selection and refinement typically span several days of iteration**

This step fits the Bayesian spatiotemporal Poisson model that links climate-induced stressors to negative-sentiment counts, using R 4.2.2 with the R-INLA, genlasso, and spdep packages.

***Note:*** Refer to fit_inla_iid_model.R and fit_inla_bym_model.R in the repository for the complete implementation.

12. Prepare data for model fitting:
  a. Compute the overall negative sentiment rate T3 = sum(NTw)/sum(TTw).
  b. Compute expected counts per observation E = TTw × T3. These will serve as the Poisson offset.
  c. Renumber indices (FID, week_idx, country_ID) to be consecutive starting from 1.
  d. Arrange data by week and region.

**CRITICAL:** Renumber FID, week_idx, and country_ID to consecutive integers starting from 1 before passing data to INLA. INLA does not validate index consistency, and gaps in the index sequence cause silent mismatches in random-effects output. Verify that length(unique(FID)) equals max(FID).

13. Build the spatial adjacency structure (optional): For BYM2 spatial model, build NUTS2 adjacency matrix using spdep::poly2nb() with queen contiguity, then convert to INLA graph format using nb2INLA().
14. Model specification: Fit the Poisson regression model in R-INLA with the following random-effect structure:
  a. Spatial random effect (IID, or BYM2 if a spatial graph is supplied), grouped by week with AR1 temporal correlation.
  b. Country-level IID random effect.
  c. Calendar-week RW1 effect for seasonality.
  d. RW1 effects for each binned climate or health covariate.
  e. Fused lasso temporal intervals (country-specific β coefficients).

**CRITICAL:** Assess multicollinearity among climate covariates before model fitting. In the associated research articles, temperature metrics exhibited |r| > 0.9, and only maximum temperature (Tmax) was retained. Users applying this protocol to a different climate dataset should run their own pairwise-correlation check and remove redundant predictors.

15. Fit the model using inla() with family=“poisson”, E=expected counts, int.strategy=“eb” for computational efficiency. Use DIC, WAIC, and CPO for model comparison.

***Note:*** The Poisson family assumes conditional variance equals conditional mean. Assess evidence of outliers, overdispersion, and general lack of fit by computing Pearson residuals and checking that they are centred near zero with no systematic structure. In Al-Ahdal et al.,^2^ residuals were mostly within ±3 with no clear trends, indicating the Poisson assumption was reasonable.

16. Extract and summarise results
  a. Extract relative risk estimates (posterior mean and 95% credible intervals) from the fitted model object.
  b. Compute Pearson residuals for model diagnostics.
  c. Extract random effects for each covariate to visualise dose–response relationships.

## Expected outcomes

Successful execution produces a fitted Bayesian spatiotemporal model from which the following outputs are extracted. Relative risk estimates for each binned covariate category are reported as posterior means with 95% credible intervals; for the categorical climate exposures (e.g., maximum temperature, drought, pollen), users should obtain a non-linear exposure–response curve across bins, with credible intervals widening at the extremes where fewer observations fall there. Random effect estimates are stored in the fitted INLA object for spatial structure (NUTS2 region), country, calendar week (seasonality), and fused-lasso temporal intervals, all then extractable from the object for visualisation as dose–response or temporal-trend plots. Model fit diagnostics including DIC, WAIC, and CPO log scores are used for model comparison, and Pearson residuals for assessing evidence of outliers, overdispersion, and general lack of fit; Pearson residuals centred near zero and mostly within ±3 without systematic temporal or spatial patterns indicate a reasonable fit (consistent with Al-Ahdal et al.^2^). Visualisation utilities for these outputs are provided in functions_plotting.R in the repository.

## Quantification and statistical analysis

Statistical analysis uses the Bayesian framework implemented in R-INLA. Posterior summaries are reported as posterior means with 95% credible intervals. Model comparison uses three criteria: DIC (Deviance Information Criterion), WAIC (Widely Applicable Information Criterion), and the log score from CPO (Conditional Predictive Ordinate). Statistical significance for random effects is assessed by whether the 95% credible interval excludes zero.

## Limitations

Geotagged tweets represent a non-random subset of social media users; typically, younger and more urban populations are represented in this sample. Results may not generalize to the whole population. In addition, this sample represents only one percent of overall tweets and this makes it less representative. LIWC22 has limitations in detecting sarcasm, irony, code-switching between languages, and cultural nuances in emotional expression. The methodology in this protocol identifies statistical associations, not causal relationships. Unmeasured confounders may influence the current observed patterns. Only a same-week associations are examined; while emotional responses to climate stressors may have different lag structures not captured by this approach. Future methodological research is needed to understand how the lagged climate effects shape the sentiments expressed on social media.

## Troubleshooting

### Problem 1

INLA model fails to converge (related to Step 15).

### Potential solution

The model may simply be too complex for the data, there can be multicollinearity among covariates, or some of the random effect levels may not have enough observations to be identifiable.

• Use IID instead of BYM2 for spatial effects to reduce complexity
• Drop any covariates that are highly correlated with one another
• Switch int.strategy=“eb” so INLA uses the faster empirical Bayes approximation
• Check that all factor levels have sufficient observations to support estimation

### Problem 2

Memory errors on HPC (related to Steps 1 and 15).

### Potential solution

Due to Insufficient RAM allocation or it is processing too many tweets simultaneously.

• Request higher memory allocation (400 GB+ recommended)
• Process data in chunks by year or country
• Increase the shell stack-size limit before launching R: at the Linux command line, run ulimit -s unlimited. This is a shell-level setting (not an INLA parameter) that prevents stack-overflow errors during large INLA model fits
• Use dtplyr for efficient data manipulation

### Problem 3

NUTS codes don’t match between datasets (related to Steps 8 and 14).

### Potential solution

NUTS classification changes between vintages (2006, 2010, 2013, 2016, 2021). Region codes and boundaries are modified over time.

• Use a consistent NUTS vintage throughout all datasets (2006 was used in the associated research articles)
• Create a crosswalk table for renamed or merged regions
• Verify that all NUTS codes in your data exist in the shapefile before modelling

### Problem 4

Language detection errors (related to Step 2).

### Potential solution

Short tweets (<10 words) produce unreliable language detection. Mixed-language tweets cause misclassification.

• Where it’s available, rely on Twitter’s native tweet_lang field as primary language indicator
• When tweet lang is missing, fall back to langdetect
• Consider excluding very short tweets (<10 words) or flagging low-confidence detections
• Verify language detection by manually checking a random sample of tweets

### Problem 5

Outliers in tweet counts (NTw, TTw) (related to Steps 1 and 7).

### Potential solution

Duplicates in the raw data these can come from bot activity, viral events, or errors introduced during aggregation.

• Look for duplicate tweets and remove them, either by tweet ID or exact text match
• Re-run aggregation after removing duplicates
• If outliers persist, examine raw tweet files for that specific region/week to identify the cause
• Verify NTw ≤ TTw for all observations
• Document whether outlier represents data error in which case fix it or real phenomenon (retain and note in limitations)

### Problem 6

Difficulty tracking files and versions (related to all steps (1–16)).

### Potential solution

Naming conventions are inconsistent across files, intermediate outputs are being overwritten, or the folder structure isn’t clear enough to keep track of what’s where.

• Establish consistent file naming: include date, step, country/region, and version (e.g., 2019_DE_sentiment_weekly_v2.csv)
• Create separate folders for raw, intermediate, and final outputs
• Never overwrite raw data files
• Maintain a processing log documenting each step and output file
• Put the analysis scripts under version control with git.

## Resource availability

### Lead contact

Further information and requests for resources and reagents should be directed to and will be fulfilled by the lead contact, Joacim Rocklöv (joacim.rocklov@umu.se).

### Technical contact

Technical questions on executing this protocol should be directed to and will be answered by the technical contact, Tareq Al-Ahdal (tareq.al-ahdal@uni-heidelberg.de).

### Materials availability

This study did not generate new unique reagents.

### Data and code availability

• All data reported in this publication will be shared by the lead contact upon request.
• Original code used in the analysis has been deposited at GitHub (https://github.com/alahdalta1/Germany_Climate_Sentiment_Paper) and archived at Zenodo (https://doi.org/10.5281/zenodo.19843730). The DOI is also listed in the key resources table.
• Any additional information required to reanalyze the data reported in this paper is available from the lead contact upon request.

## Acknowledgments

We acknowledge that the computational resources were provided by the Interdisciplinary Center for Scientific Computing (IWR) at Heidelberg University and thank the Harvard Center for Geographic Analysis for providing access to the Geotweet Archive. This work was supported by the Helmholtz Information & Data Science School for Health (HIDSS4Health) and the 10.13039/100005156Alexander von Humboldt Foundation.

## Author contributions

T.A.-A. developed the methodology, implemented the analysis pipeline, conducted all analyses with S.B., and wrote the manuscript. S.B. contributed to methodology development and manuscript review. B.A. contributed to data acquisition and processing. J.R. supervised the project, acquired funding, and reviewed the manuscript.

## Declaration of interests

The authors declare no competing interests.
